# Production of Cu/Diamond Composite Coatings and Their Selected Properties

**DOI:** 10.3390/ma17122803

**Published:** 2024-06-08

**Authors:** Grzegorz Cieślak, Marta Gostomska, Adrian Dąbrowski, Katarzyna Skroban, Tinatin Ciciszwili-Wyspiańska, Edyta Wojda, Anna Mazurek, Michał Głowacki, Michał Baranowski, Anna Gajewska-Midziałek, Maria Trzaska

**Affiliations:** 1 Łukasiewicz Research Network—Warsaw Institute of Technology, Duchnicka 3, 01-796 Warsaw, Poland; marta.gostomska@wit.lukasiewicz.gov.pl (M.G.); adrian.dabrowski@wit.lukasiewicz.gov.pl (A.D.); katarzyna.skroban@wit.lukasiewicz.gov.pl (K.S.); tinatin.ciciszwili@wit.lukasiewicz.gov.pl (T.C.-W.); edyta.wojda@wit.lukasiewicz.gov.pl (E.W.); anna.mazurek@wit.lukasiewicz.gov.pl (A.M.); michal.glowacki@wit.lukasiewicz.gov.pl (M.G.); anna.gajewska-midzialek@wit.lukasiewicz.gov.pl (A.G.-M.); maria.trzaska@wit.lukasiewicz.gov.pl (M.T.); 2 Faculty of Mechanical and Industrial Engineering, Warsaw University of Technology, Narbutta 85, 02-524 Warsaw, Poland; michal.baranowski@pw.edu.pl

**Keywords:** composite coatings, copper, nanodiamond, solderability, mechanical properties, structure

## Abstract

This article presents Cu/diamond composite coatings produced by electrochemical reduction on steel substrates and a comparison of these coatings with a copper coating without diamond nanoparticles (<10 nm). Deposition was carried out using multicomponent electrolyte solutions at a current density of 3 A/dm^2^ and magnetic stirring speed of 100 rpm. Composite coatings were deposited from baths with different diamond concentrations (4, 6, 8, 10 g/dm^3^). This study presents the surface morphology and structure of the produced coatings. The surface roughness, coating thickness (XRF), mechanical properties (DSI), and adhesion of coatings to substrates (scratch tests) were also characterized. The coatings were also tested to assess their solderability, including their spreadability, wettability of the solder, durability of solder-coating bonds, and a microstructure study.

## 1. Introduction

Composite materials are being intensively explored and developed. Synergistic interactions between the components in a composite material make the material properties better or completely new compared to individual components. In surface engineering, one of the most important techniques for coating such materials is electro-crystallization. This method provides great control over the properties of composite materials by selecting the coating metal matrix and the dispersed-phase particles (reinforcement). The properties of these materials can be controlled through process parameters, such as bath composition, deposition current density, and bath temperature. Studies have investigated various materials for reinforcing phases, incorporated into the metal matrix, such as oxides [1,2,3], nitrides [4,5], carbides [6,7,8], sulfides [9], metals [10,11], and carbon allotropes [12,13,14,15]. When embedded in the metal matrix, nanoparticles improve mechanical, tribological, or corrosion properties.

A common problem is that the dispersed phase is unevenly embedded into the coating material, resulting in inhomogeneous, anisotropic products. This issue can be countered in several ways. First, particles of appropriate shapes and sizes can be selected. Particles with nanometric dimensions and spherical shapes are generally the most favorable for deposition. Particles with other shapes, such as graphene flakes, form inhomogeneous, highly-developed surfaces [16]. Additionally, oversized particles may impede the deposition process by making it difficult to anchor such particles in the coating material [17]. Another way to achieve a more uniform embedding is to use the right mixing method, wherein the selection of the type of mixing and its speed is extremely important. Mixing plays an important role in the deposition of composite coatings by providing a constant concentration of ingredients throughout the bath, which counteracts local overheating and pitting and promotes dispersion uniformity [18]. Various methods of mixing electroplating baths have been presented in the literature, such as mechanical, magnetic, rotating electrode, ultrasonic, or compressed air [16]. The addition of a surfactant to a bath is another important factor. The literature provides many papers on the effect of different types of compounds on the embedding of particles into the metal matrix material in composite coatings [19]. 

Despite the problems encountered during the manufacturing of such materials, the potential benefits of composite coatings appear to be far more important. The continuous development of new technologies is forcing research into this type of material in electronics, where the increasing miniaturization of devices is causing problems with heat dissipation from integrated circuits. One way to prevent these components from overheating is to use materials with a suitable thermal conductivity. To date, Au and Cu have been used for coatings, while metallic matrix composites with a dispersed phase are being intensively researched to develop methods to produce materials with improved mechanical, tribological, electrical, and thermal properties. An important area of application is electronic devices. Copper is an attractive matrix material due to its good electrical and thermal conductivity. In [20], the authors reviewed the state of the art in Cu/diamond composite materials, focusing on thermal conductivity. The conclusions of the paper indicated the great potential of this type of material for applications in electronics. Another paper [21] described Cu/diamond composite layers fabricated by tape-casting and hot pressing for thermal management applications. The thermal properties of Cu/diamond composite coatings using different diamond particle sizes are presented in [22]. The thermal and electrical properties of Cu/diamond composite materials are well known, but copper exhibits a low hardness. Embedding diamond nanoparticles into the copper material is expected to improve the mechanical properties, as reported in [23,24]. These works reported an increase in the hardness of composite coatings with nickel matrix and embedded diamond compared to coatings without embedded particles. 

In this work, the main focus was to determine the effect of diamond incorporation on the structure, mechanical properties, bonding of coatings to the substrate, and the solderability of such coatings. So far, works on the mechanical properties of composite materials have focused on composites produced by various methods, including gas pressure infiltration [25], combining flake powder metallurgy and vacuum hot-press sintering [26], powder metallurgy [27,28], and cold spraying [29]. The use of chemical or electrochemical plating methods is uncommon. Examples of this can be found in [30], where a Cu/diamond composite was deposited electrochemically, but the authors used a horizontal electrode arrangement and incorporated the diamond using sedimentation. In another paper [31], copper was deposited directly onto diamond particles. The mechanical properties of electrochemically-produced Cu/diamond coatings are an area that is not fully understood. As mentioned by the authors in [32], mechanical properties are an area worthy of future research. Therefore, the present article focuses on expanding knowledge in this area, with additional emphasis on new aspects, such as the assessment of the solderability of such materials and their potential use in electronics for printed circuit boards. 

## 2. Materials and Methods

Copper (Cu) and Cu/diamond composite coatings with diamond nanoparticles as the embedded dispersed phase were deposited on S355 carbon steel substrates. Before this process, the substrate in the form of 60 × 20 × 2 mm tiles was mechanically ground on 180–1200 grit sandpaper, then degreased with acetone, activated in 15% H_2_SO_4_, and nickel-plated in a Watts bath. The deposition of Cu coatings from a sulfate bath on a steel substrate requires a sublayer to ensure good adhesion of the deposited copper coating to the substrate. The nickel plating and copper plating parameters are summarized in Table 1. A proper Cu or Cu/diamond coating was deposited onto the substrate prepared using this method.

The chemical reagents (nickel and copper salts, acids) sourced for the study from Chempur, Piekary Śląskie, Poland, were of high purity (p.a.); Cu-189 (GalwIMP, Warsaw, Poland). Diamond powder (Sigma-Aldrich, St. Louis, MO, USA) with a particle size of less than 10 nm was used as the dispersed phase. The diamond was characterized by using X-ray diffraction (XRD). The diamond used was artificially synthesized. Diamond nano-powder can be produced by multi-cathode chemical vapor phase deposition in DC plasma and high pressure high temperature (HPHT). Potential applications for diamond nano-powder include biosensors. Surface modification of the nanodiamond can improve its mechanism of bonding to specific biomolecules [33]. In this study authors used diamond powder that was not functionalized or modified in any way (despite the lack of wettability of diamond with respect to copper), and the incorporation of the dispersion phase took place by electrochemical reduction.

The coatings were deposited at a current density of 3 A/dm^2^, a stirring rate of 100 rpm, using copper anodes, and a bath temperature of 298 K ± 2 K. The list of samples and labels is summarized in Table 2.

The morphology and surface topography of the produced coatings were characterized using a scanning electron microscope (FE Zeiss Merlin, Carl Zeiss AG, Oberkochen, Germany). The thickness of the produced coatings was measured using a Fischerscope XRAY XDV–SDD X-ray fluorescence spectrometer (Helmut Fischer, Sindelfingen, Germany). The number of dispersed-phase particles in the composite coatings was weighed by dissolving the Cu/diamond films produced earlier and weighing the remaining diamond particles. The mechanical properties of the produced coatings were studied using depth-sensing indentation (DSI) on metallographic polished sections made on cross-sections perpendicular to the surface. Samples were prepared by cutting off a 5 mm sample and putting it in thermosetting resin (Struers). Then, samples were ground with 120–1200 grit sandpaper and polished with diamond paste. The crystal structure of the coatings was examined by X-ray diffraction (XRD; Rigaku Mini Flex II) at a 2θ range of 20–120° using Cu-kα radiation (*λ* = 1.5418 Å) on an area of 100 mm^2^. The roughness parameters Ra and Rz were measured using a Surftest SJ210 profilometer (Mitutoyo, Kawasaki, Japan). The adhesion of the produced coatings onto the substrate was tested by scratch test using a CSEM Revetest device (Anton Paar GmbH, Graz, Austria) with a progressive load of 0–100 N for 60 s at a Rockwell indenter travel speed of 10 mm/min. 

**Figure 1:**
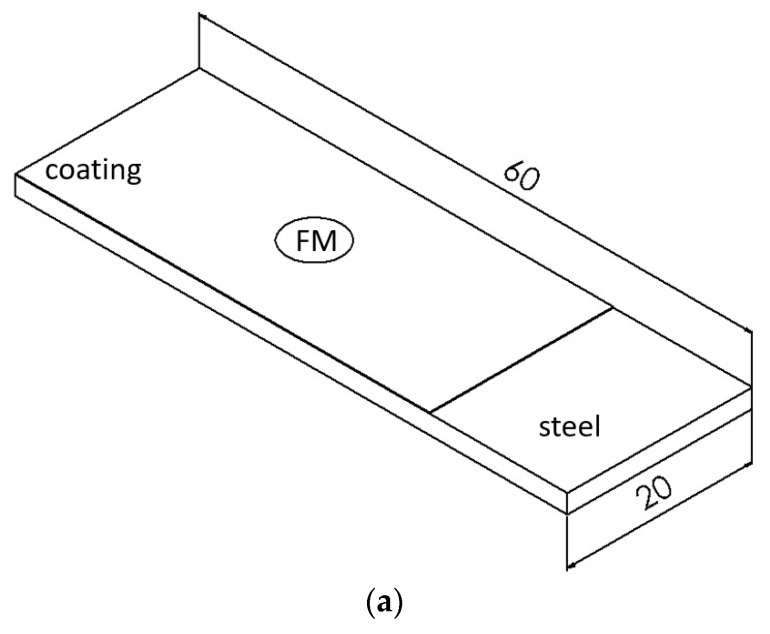

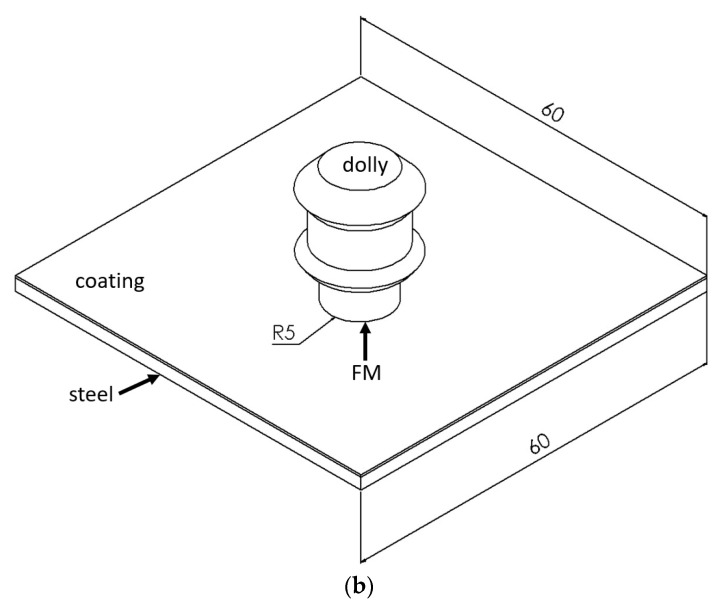
Samples (**a**) for spreadability and microstructure tests (substrate-coating-solder), (**b**) for adhesion test. (unit: mm).

Technological properties were determined in terms of suitability for soldering in electronics. The study investigated the interaction of Cu/diamond coatings with three solder alloys (FM): SnCu1, SAC 305, and Sn60Pb40, which differed in their chemical composition and melting point (Table 3). Solders were applied in the form of wires with a diameter of ⌀1.5 mm. Zinc chloride-based flux was used during sample production. The study included two types of samples. The first type, in which a steel substrate was covered with electroplated coatings (Cu or Cu/diamond-10 g/dm^3^), on which solder formed droplets (Figure 1a), was used for spreadability and microstructure tests. In the second type of sample, dollies made of electrolytic copper were soldered onto the electroplated substrate (Figure 1b), which enabled the adhesion test. The sample with the highest concentration of diamond in the bath was selected for testing.

Solder spreadability tests on coatings, microstructure tests of the solder-coating interaction zone, and an adhesion test for solder-coating joints were performed. After the spreadability tests, observations were made using an Olympus SZ61 stereoscopic microscope. Using Olympus Stream Essentials 2.5.2 software, the surface areas of all the droplets were determined to perform a spreadability analysis. In samples tested for spreadability, the weight of each solder was calculated from its specific gravity [34], assuming the same volume of 34 mm^3^ for each variant. During sample fabrication, materials were heated above the liquidus of individual solders. The interaction time between the liquid solder and the coating for each variant was 15 s. Metallographic polished sections were made from the substrate-coating–solder samples and observed using an optical microscope. Using the Keyence VHX-5000 digital microscope (Keyence, Osaka, Japan), we focused on areas of contact between the liquid solder and solid coating under a gaseous atmosphere. During these microscopic observations, contact angles were measured. The solder-coating contact zone was observed to determine the chemical composition by EDX using a Hitachi SU70 scanning electron microscope. For these observations, the sample surface was prepared using an ion polisher. An Elcometer 510 automatic coating adhesion tester was used to test the strength of the bond between the solder and the coating. During the tests, a ø10 mm dolly was used, which provided a measuring range of 8–100 MPa and used the pull-off method. Tension was incremented by 0.8 MPa/s during the test.

## 3. Results and Discussion

### 3.1. Structure of Diamond and Produced Coatings

**Figure 2:**
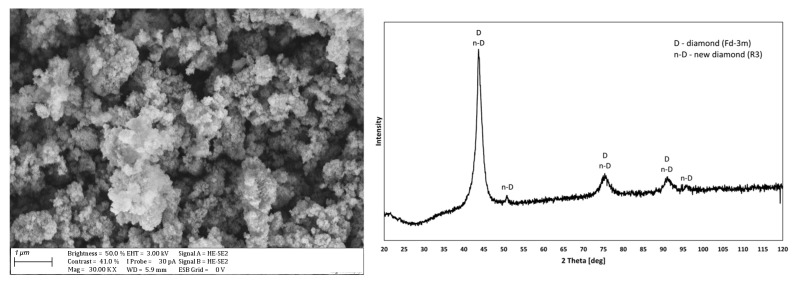
Surface morphology and XRD pattern of the used diamond.

**Figure 3:**
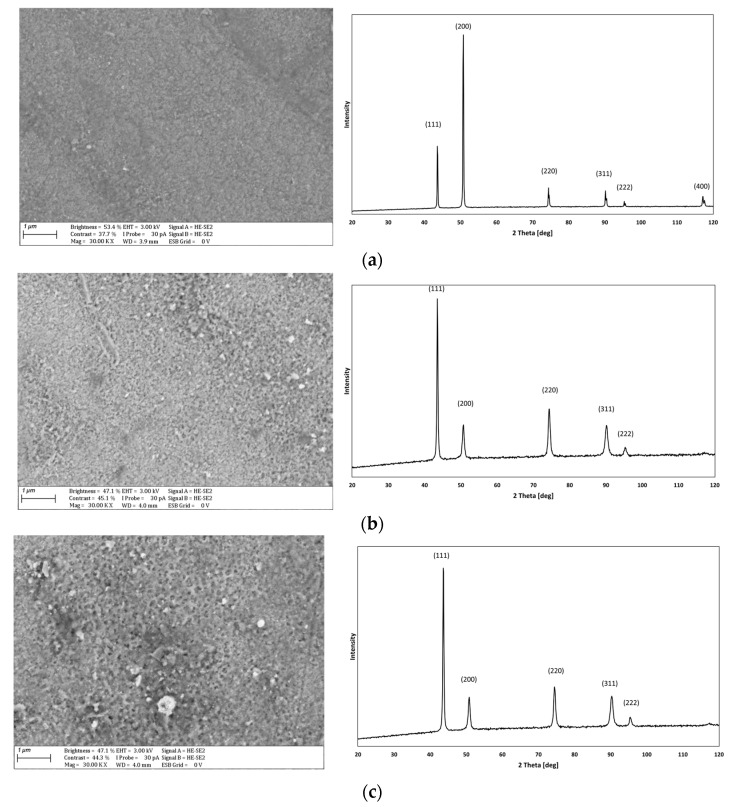

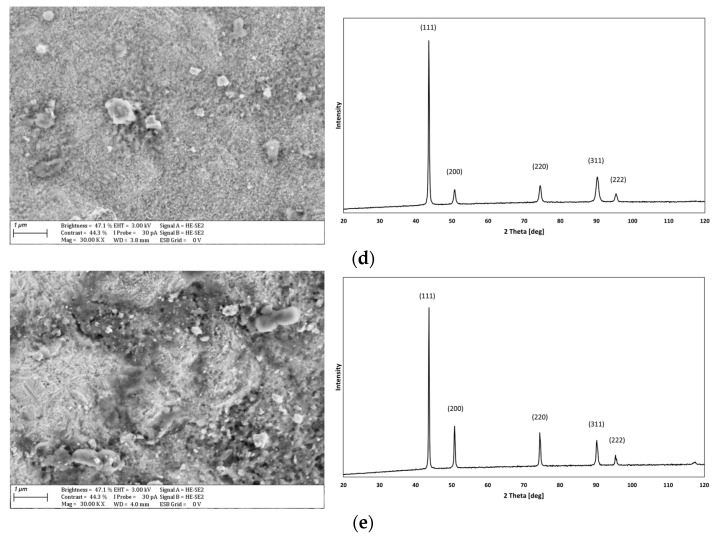
Surface morphology and XRD patterns of produced coatings: (**a**) Cu; (**b**) Cu/diamond (4); (**c**) Cu/diamond (6); (**d**) Cu/diamond (8); (**e**) Cu/diamond (10); ICDD 00-004-0836.

Figure 2 shows the surface morphology and XRD pattern of the analyzed diamond powder. It shows that it may be a new diamond phase with an R3-type structure (according to ICDD card 01-075-9130) or a mixture with diamond of known structure Fd-3m (according to ICDD card 00-006-0675). The tested material tended to form agglomerates, which is a typical phenomenon for diamond nanoparticles commonly sold as suspensions or powders. Mochalin et al. [35] mentioned the difficulty of breaking such structures into single particles and also noted that the results of experiments with such materials related more to agglomerates than to single diamond nanoparticles. Images of the surface morphology and XRD patterns of the tested Cu and Cu/diamond composite coatings are shown in Figure 3. 

In the Cu coating without embedded particles, the surface was smooth and shiny, which is characteristic of copper coatings deposited from baths containing organic additives [15,36]. Upon increasing the concentration of the dispersed phase in the bath, the surface morphology and thickness of the Cu/diamond composite coatings changed noticeably. This was due to the incorporation of nanoparticles and agglomerates of the dispersed phase into the copper matrix, which reduced the efficiency of the deposition of Cu/diamond composite coatings. The surface morphology of the composite coatings underwent typical changes that have been presented in [12,15,16]. These changes are well reflected in the measurements of roughness parameters and surface thickness of the tested coatings (Table 4). 

For the tested coatings, there was a significant increase in the roughness parameters Ra and Rz when using a dispersed-phase bath concentration of 10 g/dm^3^. Multiple studies on the concentration of the dispersed phase in the bath have shown that, for each type of particle, there are critical concentrations at which the deposited coatings have the best properties. If these concentrations are exceeded, it adversely affects the properties of manufactured composite coatings [37,38]. 

The co-deposition of a metal matrix and dispersed particles has been studied for many years, but it remains difficult to identify a single dominant mechanism of co-deposition. It is influenced by too many variables and should be considered separately for each type of particle, bath, and deposition parameters. The impact of parameters and selected mechanisms of co-deposition have been addressed in [39]. The authors describe the effects of various parameters on the co-deposition of dispersion particles and metal matrix. In addition, they list some of the best-known mechanisms, but each of them is somewhat simplified and can only be considered in specific cases. 

The incorporation of diamond nanoparticles into a copper matrix affects the morphology and, more significantly, also the structure of the materials produced. In the case under study, the matrix was copper with a crystalline structure. Diffraction studies of the produced Cu and Cu/diamond coatings (Figure 3) indicated changes in structure, especially in the favored direction of crystallite growth. For Cu coating without embedded particles, the <200> direction was dominant, while the <111> direction was most intense for composite coatings. This may be due to the incorporation of diamond into the Cu matrix. The reflections for diamond overlapped with those of copper, possibly resulting in their higher intensity in the composite coatings. This parameter can have a major impact on the properties of composite coatings. The maximum unbalance between directions <111> and <200> for sample on Figure 3e corresponds to the amount of embedded diamond in the matrix material Cu (Table 5). The much higher degree of surface development compared to other coatings and the greater tendency to form agglomerates may be the reason for the lower peak asymmetry for the Cu/diamond coating (10). In this aspect, the mixing method seems to be an important parameter to prevent agglomeration. In [40], Ni/diamond coatings were deposited using ultrasonic agitation. This allowed coatings with a more even incorporation of diamond particles to be obtained. 

The dispersed phase embedded into the composite coating was weighed by dissolving the copper matrix material, and the results are shown in Table 5. 

Weight measurements indicated that diamond accounted for 1.1–1.8% of the coating’s weight, depending on the concentration in the bath after embedding. Even such small amounts seemed to affect the structure and properties of the Cu/diamond composite coatings produced. At higher concentrations of the dispersed phase, dispersed phase agglomerates were formed in the aqueous environment of the bath, which were then incorporated into the copper matrix.

### 3.2. Mechanical Properties of Produced Coatings

**Figure 4:**
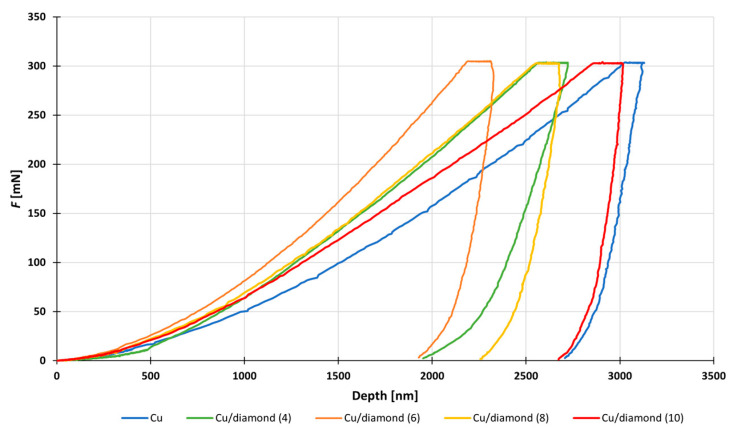
Dependence of load on the depth of penetration of the indenter into the material of the tested Cu and Cu/diamond coatings.

Material properties are extremely important and depend on their application, but mechanical properties are an important group for all materials. As part of the study, microhardness (Martens, indentation, Vickers), modulus of elasticity, and elastic strain were determined, and the results are shown in Figure 4 and Table 6.

The loading and unloading curves were used to determine material parameters in the Cu and Cu/diamond composite coatings examined. The embedding of nanodiamond into the copper matrix affected the mechanical properties of the Cu/diamond composite coatings. All variants of composite coatings with embedded nanodiamond showed better mechanical properties compared with copper coatings without embedded particles. The highest hardness was obtained for the coating deposited from a bath with a diamond concentration of 6 g/dm^3^. This was illustrated by the indentation depth into the coating during the test (Table 6). The coating material deposited from a bath with a diamond concentration of 6 g/dm^3^ exhibited the greatest resistance when the indenter was pressed against it with a progressive load. The greatest indentation depth was achieved for the copper coating without embedded dispersed phase particles. In this type of material, several mechanisms may play a role in reinforcement. The first is dispersive reinforcement (Orowan mechanism), according to which the particles introduced into the matrix block the movement of dislocations, resulting in a strengthening of the material. Another is the fragmentation of the structure according to the Hall–Petch relationship. Introduced particles contribute to blocking the growth of metal matrix crystallite and the formation of new crystallization centres. As a result, the proportion of grain boundaries increases, which strengthens the material. In [12], the authors explained the increase in hardness of composite coatings with embedded diamond by dispersion reinforcement and the properties of diamond, which was characterized by very high hardness. Also significant was the effect of changes in the crystallographic orientation of Cu/diamond composite coatings compared with a copper coating without embedded dispersed phase particles. The results obtained indicate that the use of diamond as a dispersion phase can contribute to improved mechanical properties. This is also confirmed by studies on coatings with other matrices, where the incorporation of diamond contributed to improved mechanical and tribological properties in Ni-W [41] and Ni-P [42] coatings. Deng et al. [43] produced Ni/diamond coatings for slicing blade applications. The addition of diamond increased hardness and wear resistance with a high diamond content embedded in the coating.

### 3.3. Scratch Test 

**Figure 5:**
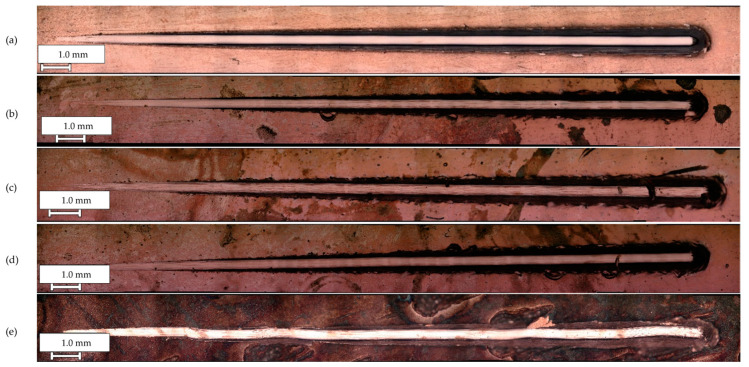
Images of scratches after scratch testing: (**a**) Cu; (**b**) Cu/diamond (4); (**c**) Cu/diamond (6); (**d**) Cu/diamond (8); (**e**) Cu/diamond (10).

**Figure 6:**
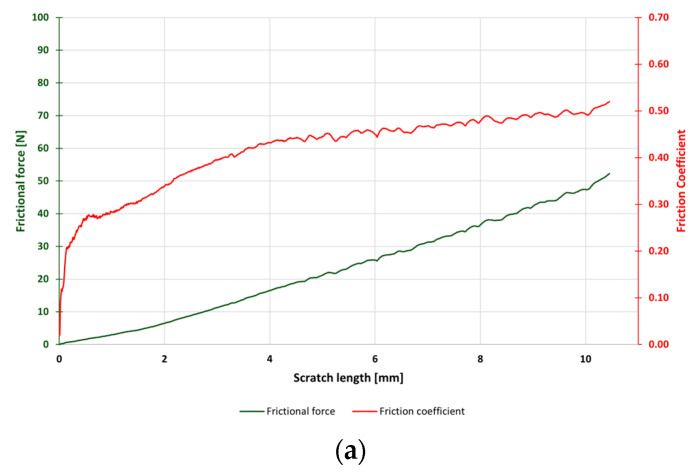

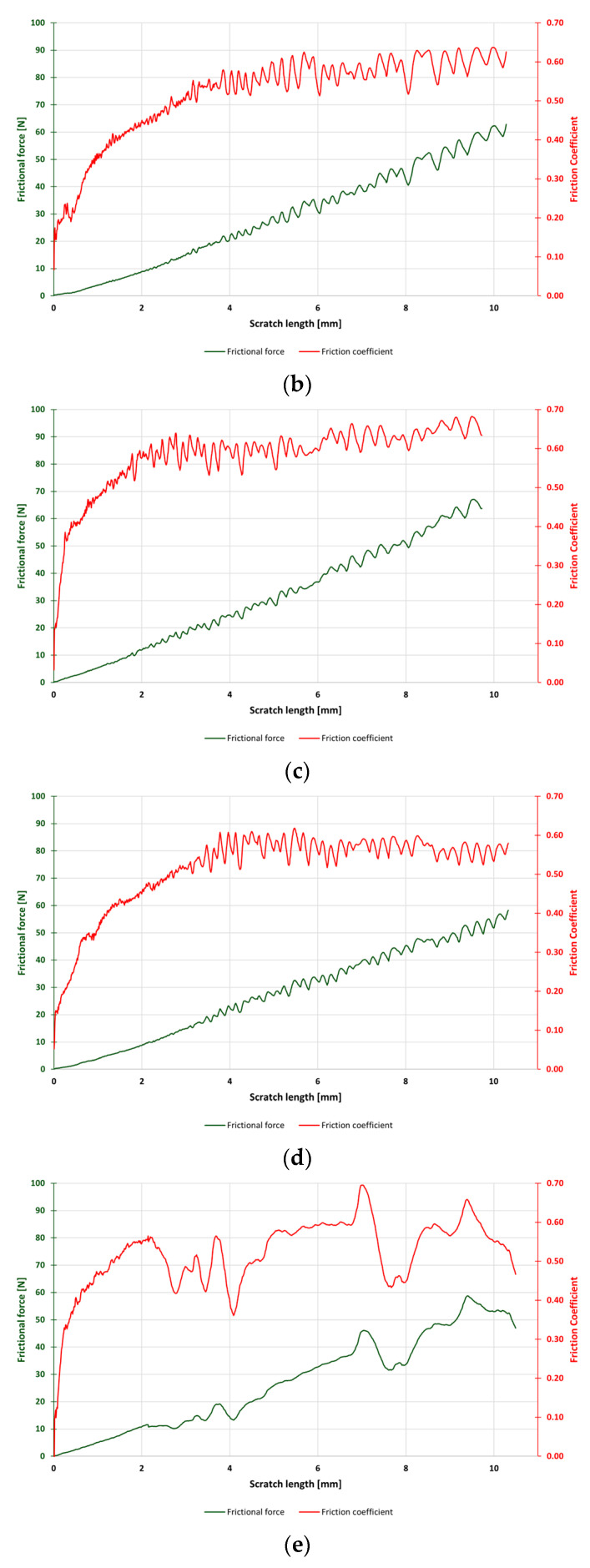
Frictional force and friction coefficient during the scratch test of produced coatings: (**a**) Cu; (**b**) Cu/diamond (4); (**c**) Cu/diamond (6); (**d**) Cu/diamond (8); (**e**) Cu/diamond (10).

Scratch tests were used to determine the adhesion of the produced Cu and Cu/diamond coatings with the steel substrate. Using a Rockwell-type indenter, scratches were made on the surface of the tested coatings by applying a progressive load. The results of the tests were critical load values *L_c1_* and *L_c2_*, as summarized in Table 7. Force diagrams during the test and damage images are shown in Figure 5 and Figure 6.

The results exhibited similar damage to all coatings. Upon increasing the load, elastic deformation initially occurred on the surfaces of the tested samples, followed by plastic deformation and, finally, cracking of the coating. In the case of the tested materials, the first cohesive cracks formed at the critical load *L*_c1_. This type of damage occurred due to friction between the moving indenter and the tested coating and was the result of stress concentration in front of and behind the indenter [44]. Typical examples of damage of this type were cracks perpendicular to the direction of indenter movement. In the case of the copper coating without embedded particles, the first oblique cracks from the center of the scratch were observed at a load of 12.85 N and continued to grow until the coating material lapped at the end of the scratch. In the case of Cu/diamond composite coatings, agglomerates of the embedded dispersed phase made it difficult to accurately locate the initial damage. For Cu/diamond coatings, cohesive damage occurred at *L_c_*_1_ between 20 N and 30 N in the form of longitudinal cracks, possibly indicating delamination. In the further section of the scratch, these phenomena were more severe, eventually manifesting as damage in the form of cracks with chipping, peeling, and lapping of the coating material under *L*c_2_ loads of approximately 80 N. The Cu/diamond coating (10) showed a high degree of surface development and the smallest thickness, and crack waving was observed during the scratch test. 

### 3.4. Solderability of Tested Coatings

**Figure 7:**
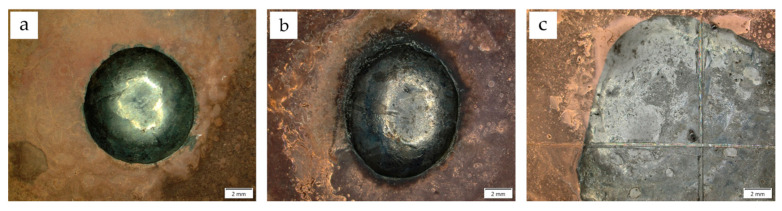
Solder spreadability on Cu coating: (**a**) SnCu1 solder alloy, (**b**) SAC305 solder alloy, (**c**) Sn60Pb40 solder alloy.

**Figure 8:**
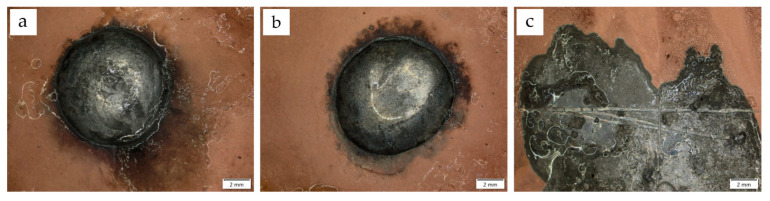
Solder spreadability on Cu/diamond (10) coating: (**a**) SnCu1 solder alloy, (**b**) SAC305 solder alloy, (**c**) Sn60Pb40 solder alloy.

To characterize the technological properties (solderability), three different solder alloys were selected, which are commonly used for copper bonding, taking into account their applicability in electronics. The results of the spreadability tests are presented in the form of macroscopic images (Figure 7 and Figure 8). The values of the measured areas of all droplets are shown in Table 8.

**Figure 9:**
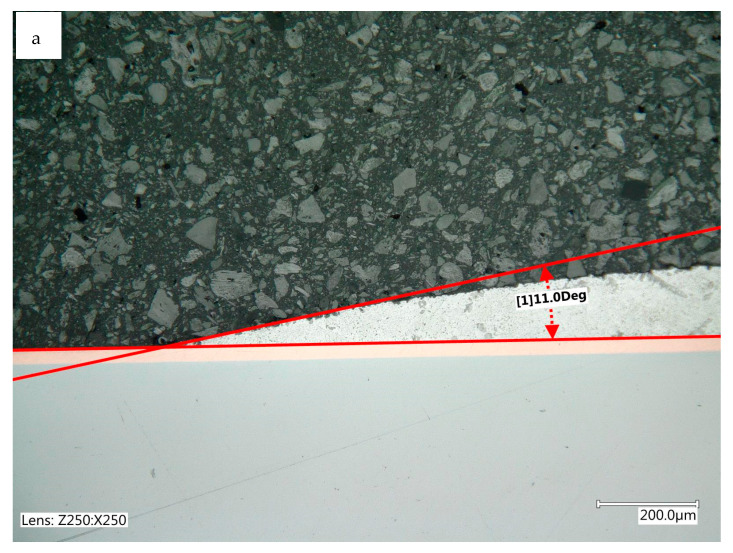

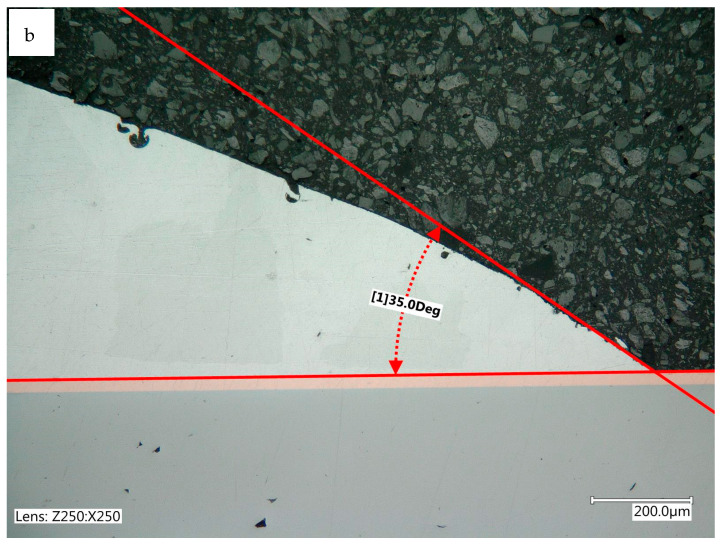
Selected contact angle measurements: (**a**) Sn60Pb40 solder alloy Cu/diamond (10) coating; (**b**) SnCu1 solder alloy Cu/diamond (10) coating.

The formation of a soldered joint and its quality depend on wettability and spreadability, defined as the ability of a liquid solder to form a thin, even, and continuous coating that covers a surface. Evaluating the FM’s spreadability over the surface of the soldered component and wettability are important indicators of solder suitability [45,46]. For the tested coatings, the addition of diamond did not significantly reduce their ability to be coated with liquid solder. Compared with Cu coatings, the spreadability and wettability parameters were similar for each solder. The spreadability was determined after the solder crystallized. The Sn60Pb40 solder, used as a reference material in the tests owing to the presence of lead (RoHS directive), had by far the best spreadability. The main limitation of the spreadability of this material was the volume of solder used. A very small wetting angle was obtained for this solder (Figure 9a). Values around 10° indicated the very good solderability of Sn60Pb40 coatings.

SnCu1 and SAC305 solders have replaced Pb materials in industrial applications for soldering electronic components. Relatively smaller surface areas were obtained for these solders, but the results still indicate the good spreadability of the additives on the surfaces of copper–diamond coatings. The spreadability of the SAC305 solder was slightly better than that of SnCu1. The contact angles of Cu/diamond (10) coatings (Figure 9b) indicated their good solderability. With values of around 38°, no processing problems during the formation of joints are anticipated [47].

**Figure 10:**
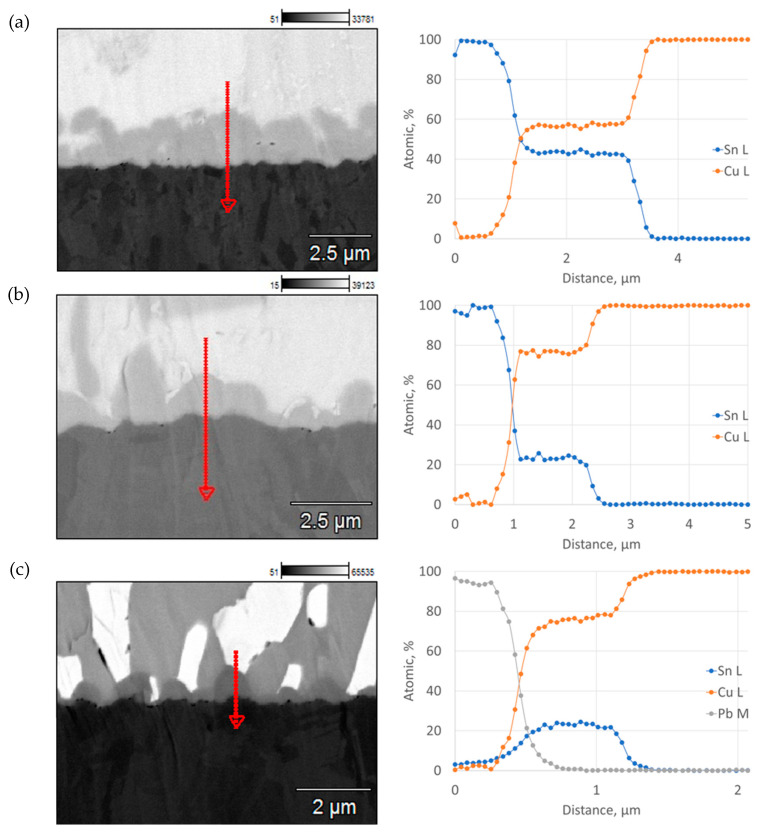
Linear distribution of elements in the contact zone between solder and Cu coating: (**a**) SnCu1; (**b**) SAC305; (**c**) Sn60Pb40.

**Figure 11:**
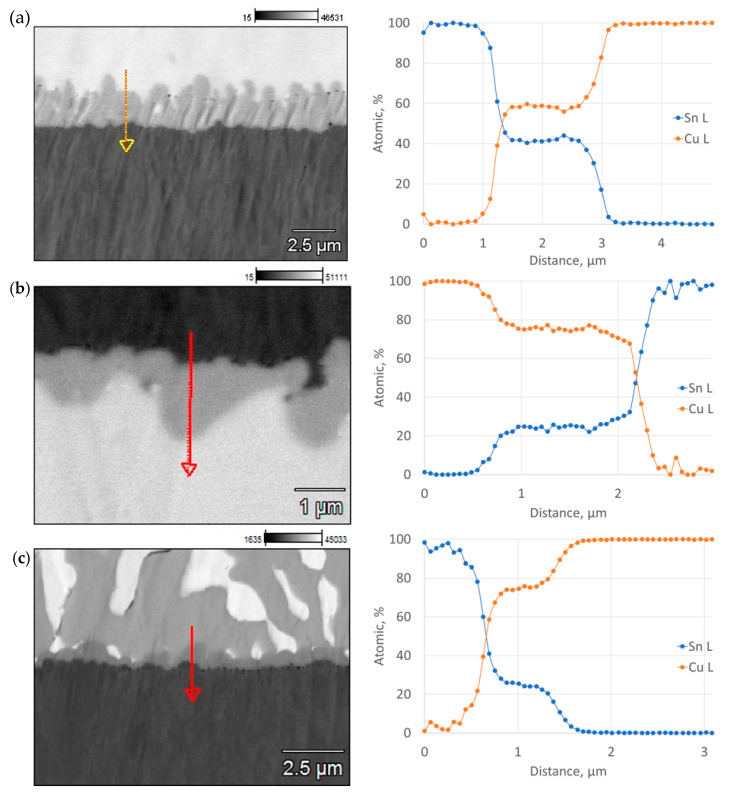
Linear distribution of elements in the contact zone between solder and Cu/diamond (10) coating: (**a**) SnCu1; (**b**) SAC305; (**c**) Sn60Pb40.

For all variants, the observations of the FM–coating interaction zone confirmed that new phases were formed at their interface, which allowed their thickness and chemical composition to be measured (Figure 10 and Figure 11). 

The microstructure of soldered joints at the interface between coating and FM was analyzed to observe how intermetallic phases were formed, which differed in chemical composition and thickness for each variant. Depending on the solder, the thickness of these phases ranged from about 3 µm (SnCu1) to values below 1 µm (Sn60Pb40). The results of the chemical composition analysis indicated that for SnCu1, solder, a Cu6Sn5 phase formed at the FM–coating interface. However, for SAC305 and Sn60Pb40 solders, a Cu3Sn phase was formed [48,49]. 

**Figure 12:**
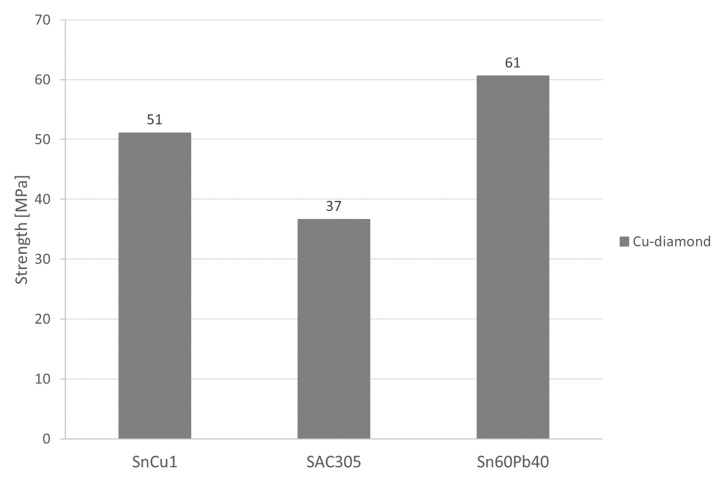
Results of the adhesion test.

The solder–coating bonds were tested for their strength using an adhesion test. For all variants, pull-off occurred in the solder. The stress required to detach each soldered dolly from the Cu/diamond coating is shown in Figure 12.

The contact angle is responsible for the interaction of the solder with the coating. The results of the adhesion test indicate lower solder alloy strength.

## 4. Conclusions

The research objects were composite coatings with a copper matrix and embedded nanodiamond as the dispersed phase. The resulting coatings showed compact structures and good bonding to the steel substrate. At nanodiamond bath concentrations of 4–10 g/dm^3^, 1–2% of the dispersed phase was incorporated into the composite coating. Even such a low value significantly affected the structure and properties of the produced coatings. The incorporation of diamond nanoparticles and agglomerates changed the morphology and topography of the surface, as shown by SEM images and roughness parameters. The introduction of the dispersed phase also affected the structure of the materials studied by changing the preferred growth direction of the crystallites of the matrix material (Cu) from the <200> to the <111> direction. Cu/diamond composite coatings had a higher hardness than copper coatings without embedded diamond nanoparticles. For the deposited composite coatings, there were optimum bath dispersed phase concentrations, at which the coating properties were the best and above which the material properties deteriorated. For the series of samples tested, the highest hardness was achieved for the variant deposited from a bath with a dispersed phase concentration of 6 g/dm^3^. The technological properties (solderability) investigated in this paper allowed for the selection of FM that enabled the soldering of Cu/diamond coatings. Because the solder substrate showed wetting angles below 40° in Cu/diamond composite coatings, no technological problems are expected during joint formation. The research carried out in this work indicated that nanodiamond as a dispersed phase embedded in a copper coating shows great potential for electronics applications, particularly in printed circuit boards. However, the properties of the materials obtained depend more on the agglomerates than on the individual nanoparticles. Future research into this type of material should focus on the dispersion characteristics and their effect on the resulting properties.

## Figures and Tables

**Table 1:** Bath compositions and process parameters for deposition of Ni sublayer and Cu and Cu/diamond composite coatings.

Process	Bath Composition	Process Parameters
Nickel plating	NiSO_4_*7H_2_O p.a., NiCl_2_*6H_2_O p.a., H_3_BO_3_ p.a.	3 A/dm^2^, 100 rpm, temp. 318 K
Copper plating	CuSO_4_*5H_2_O p.a., H_2_SO_4_ p.a., HCl p.a., Cu-189, diamond (0/4/6/8/10 g/dm^3^)	3 A/dm^2^, 100 rpm, temp. 298 K

**Table 2:** List of samples and labels.

Coating	Concentration of Diamond Particles in the Bath (g/dm^3^)
Cu	0.00
Cu/diamond (4)	4.00
Cu/diamond (6)	6.00
Cu/diamond (8)	8.00
Cu/diamond (10)	10.00

**Table 3:** Chemical composition and melting point range of solder alloys [34]

Solder	Composition (%)				Melting Point Range (°C)
	Sn	Ag	Cu	Pb	
SnCu1	99	-	1	-	230–240
SAC 305	96.5	3	0.5	-	217–219
Sn60Pb40	60	-	-	40	183–190

**Table 4:** Surface roughness and thickness of the tested Cu and Cu/diamond coatings.

Coating	Roughness Parameter		Thickness (µm)
	Ra (µm)	Rz (µm)	
Cu	0.04 ± 0.01	0.38 ± 0.13	30.6 ± 2.4
Cu/diamond (4)	0.21 ± 0.03	1.70 ± 0.26	28.1 ± 1.6
Cu/diamond (6)	0.31 ± 0.04	2.32 ± 0.48	26.0 ± 1.8
Cu/diamond (8)	0.44 ± 0.11	3.10 ± 0.98	25.7 ± 1.4
Cu/diamond (10)	4.89 ± 0.53	26.22 ± 2.28	17.0 ± 1.2

**Table 5:** Diamond content in the form of dispersed particles in composite coatings.

Concentration of Diamond Particles in the Bath (g/dm^3^)	Weight of Cu/Diamond Coating (g)	Weight of Embedded Diamond Particles in Cu/Diamond Coating (g)	Content of Diamond in the Coating (%)
0	0.673 (±0.047)	0	0
4	0.652 (±0.001)	0.009 (±0.000)	1.3
6	0.667 (±0.004)	0.007 (±0.000)	1.1
8	0.639 (±0.017)	0.012 (±0.001)	1.8
10	0.658 (±0.005)	0.010 (±0.001)	1.6

**Table 6:** Mechanical properties of Cu and Cu/diamond coatings from DSI tests.

Coating	Microhardness			Modulus of Elasticity EIT (GPa)	Maximum Penetration Depth (nm)
	HIT (MPa)	HM (MPa)	HV		
Cu	1439 (±26)	1197 (±21)	135 (±3)	82 (±13)	3094 (±28)
Cu/diamond (4)	2076 (±32)	1534 (±23)	196 (±3)	52 (±2)	2737 (±20)
Cu/diamond (6)	2644 (±24)	2164 (±23)	250 (±2)	132 (±5)	2305 (±14)
Cu/diamond (8)	1993 (±96)	1644 (±84)	188 (±9)	105 (±9)	2643 (±71)
Cu/diamond (10)	1560 (±62)	1340 (±50)	147 (±6)	128 (±4)	2927 (±56)

**Table 7:** Critical load values for tested Cu and Cu/diamond composite coatings.

Coating	Critical Load *L_c_*_1_ (N)	Critical Load *L_c_*_2_ (N)
Cu	12.85	62.30
Cu/diamond (4)	30.97	78.45
Cu/diamond (6)	22.83	81.46
Cu/diamond (8)	31.99	85.37
Cu/diamond (10)	22.49	73.31

**Table 8:** Spreadability test showing results of droplet area measurements.

Coating	Area of Solder Droplet (mm^2^)		
	SnCu1	SAC305	Sn60Pb40
Cu	59.92	71.55	320.10
Cu/diamond (10)	64.46	68.12	282.84

## Data Availability

The raw data supporting the conclusions of this article will be made available by the authors on request.
